# Direct *N*-substituted *N*-thiocarboxyanhydride polymerization towards polypeptoids bearing unprotected carboxyl groups

**DOI:** 10.1038/s42004-020-00393-y

**Published:** 2020-10-28

**Authors:** Botuo Zheng, Tianwen Bai, Jun Ling, Jihong Sun

**Affiliations:** 1 Department of Radiology, Sir Run Run Shaw Hospital, School of Medicine, Zhejiang University, Hangzhou, 310016 China; 2 MOE Key Laboratory of Macromolecular Synthesis and Functionalization, Department of Polymer Science and Engineering, Zhejiang University, Hangzhou, 310027 China; 3 Innovation Center for Minimally Invasive Techniques and Devices, Zhejiang University, Hangzhou, 310016 China

**Keywords:** Polymer synthesis, Bioinspired materials

## Abstract

Synthesis of poly(α-amino acid)s bearing carboxyl groups is a critical pathway to prepare biomaterials to simulate functional proteins. The traditional approaches call for carboxyl-protected monomers to prevent degradation of monomers or wrong linkage. In this contribution, we synthesize *N*-carboxypentyl glycine *N*-thiocarboxyanhydride (CPG-NTA) and iminodiacetic acid *N*-thiocarboxyanhydride (IDA-NTA) without protection. Initiated by amines, CPG-NTA directly polymerizes into polyCPG bearing unprotected carboxyl groups with controlled molecular weight (2.8–9.3 kg mol^−1^) and low dispersities (1.08–1.12). Block and random copolymerizations of CPG-NTA with *N*-ethyl glycine *N*-thiocarboxyanhydride (NEG-NTA) demonstrate its versatile construction of complicated polypeptoids. On the contrary, IDA-NTA transforms amines into cyclic IDA dimer-capped species with carboxyl end group in decent yields (>89%) regio-selectively. Density functional theory calculation elucidates that IDA repeating unit is prone to cyclize to be the six-membered ring product with low *ΔG*. The polymer is a good adhesive reagent to various materials with adhesive strength of 33–229 kPa.

## Abstract (web summary)

Synthetic polymers bearing carboxylic acid pendants typically require the use of protecting group transformations to access. Here direct polymerization of N-carboxypentyl N-thiocarboxyanhydride derivatives offers access to carboxypolypeptide analogs while avoiding undesired side reactions.

## Introduction

Functional α-amino acids play essential roles in life activities by constituting active sites of proteins and sustaining elegant architectures^1–3^. α-Amino acids bearing carboxyl groups, such as glutamic acid and aspartic acid, attract great attention because of their reactivity, pH-responsiveness and ability to chelate metal ions^4–6^. Scientists have made great progress in synthesizing acidic polypeptides to realize applications including self-assembly, biopharmaceutics, food industries, hydrogels and biosensors^7–22^. Their analogs, *N*-carboxyalkyl polypeptoids, are also developed as promising building blocks for self-assembly structures and functional materials^23–27^ thanks to their excellent design flexibility and processability compared with polypeptides.

Although techniques have been developed to prepare acidic polypeptides and polypeptoids, it is still a strenuous and challenging work to introduce amino acid repeat unit with carboxyl groups into the polymer especially in the system containing a variety of functional groups^28^. The most popular approaches are ring-opening polymerization (ROP) of α-amino acid *N*-carboxyanhydride (NCA)^29–36^ and solid phase peptide synthesis (SPPS)^37–39^. NCAs are high reactive and able to prepare ester-protected poly(glutamic acid), poly(aspartic acid) and other polypeptides bearing inert groups with high molecular weights (MWs)^15,40,41^. Extra steps are required to introduce intact carboxyl groups by deprotection or transformation reaction from side groups such as vinyl and thioester^28^. The protecting benzyl ester groups also suffer from the risk of aminolysis by amine end groups^42^. Although SPPS does well in synthesizing sequence-defined poly(α-amino acid)s containing carboxyl group, it can only be carried out in milligram scales^37^, and esterification protection of carboxyl side group is also necessary^24,25^ to prevent its wrong amidation with propagating amine group. In traditional ways, the tedious deprotection and post-modification steps take scientists great effort and time in design and purification leading to loss of products.

α-Amino acids *N*-thiocarboxyanhydrides (NTAs) are more stable monomers than NCAs^43–46^. Our group has reported that NTAs and their polymerizations tolerate mercaptans^47^, alcohols^48^, phenols^49^ and water^50,51^. Zhang et al. further proved that weak organic acid is not inhibitor or pollutant but catalyst of NTA polymerization^52^. The polymerization of β-amino acid NTAs was also found an effective way to synthesize β-peptides^53^. However, NTAs with carboxyl groups are barely investigated^54,55^, and their polymerizations have never been achieved to the best of our knowledge.

In this contribution, we synthesize two unprotected *N*-substituted NTAs (NNTAs), i.e., *N*-carboxypentyl glycine NTA (CPG-NTA) and iminodiacetic acid NTA (IDA-NTA) from aminocaproic acid and iminodiacetic acid both of which are extensively commercially available from industry. CPG-NTA shows good reactivity in the direct polymerization into polyCPGs with high MWs and low dispersities. On the contrary, instead of polymerization, IDA-NTA undergoes amidation of amine end group regio-selectively and transforms amines into carboxyl-ended species while hydroxyl groups keep intact. Detailed mechanism is further revealed by density functional theory (DFT) calculation. The polymerization of *N*-carboxybutyl glycine NTA (CBG-NTA) demonstrates that CPG-NTA is not an isolated case of polymerizable NNTA monomer with unprotected carboxyl groups. Physical property and pH-responsiveness of polyCPG are investigated and polyCPG serves as a good adhesive for various materials.

## Results and discussion

### Preparation and polymerization of CPG-NTA

**Fig. 1:**
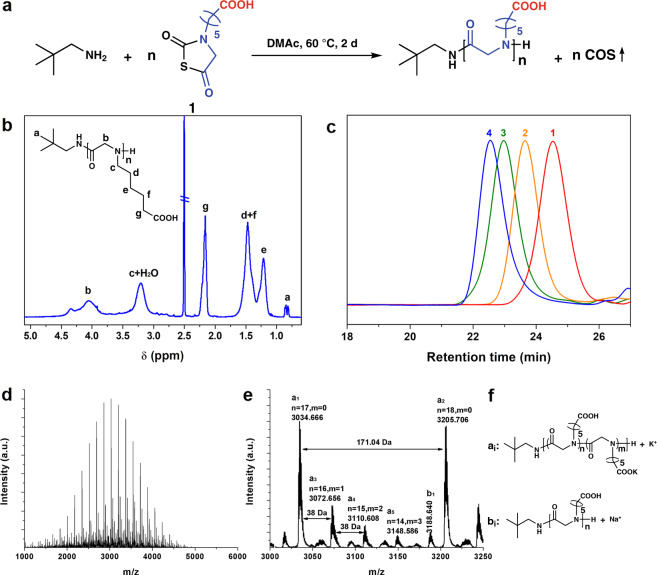
Polymerization of CPG-NTA and characterization of polyCPGs. The scheme of CPG-NTA homo-polymerization and block copolymerization with NEG-NTA initiated by neopentylamine (**a**). ^1^H NMR spectrum of polyCPG (Sample 3, // dimethyl sulfoxide (DMSO)) (**b**), SEC traces of product obtained from CPG-NTA polymerization (Samples 1–4) (**c**) and MALDI-ToF MS of polyCPG (Sample 2) (**d**) with the zoom-in view (**e**) and the corresponding chemical structures (**f**).

Aminocaproic acid is extensively accessible by the hydrolysis of caprolactam and recycled Nylon 6. Incorporation of aminocaproic acid as a submonomer into polypeptoids is an effective and noteworthy way to utilize it. From aminocaproic acid we successfully synthesize CPG and CPG-NTA (**1** in Fig. 1a, Supplementary Fig. 1). CPG-NTA is characterized by ^1^H nuclear magnetic resonance (^1^H NMR, Supplementary Fig. 2a), ^13^C NMR (Supplementary Fig. 2b) and electrospray ionization mass spectra (ESI-MS, Supplementary Fig. 3). In its ^1^H NMR spectrum, the signal ascribed to carboxylic acid proton is observed at the chemical shift of 11 ppm. Three carbon signals at 194.18, 179.50 and 165.04 ppm in ^13^C NMR indicate the existence of three carbonyl groups with various chemical environments rather than two typical carbonyl signals of other NNTAs reported^45^. CPG-NTA with unprotected carboxyl groups is stable in purification and storage for months.

Direct polymerizations of CPG-NTA succeed without the protection on carboxyl group (Fig. 1a) as summarized in Table 1. *N,N*-dimethylacetamide (DMAc) is a good solvent for both acidic polymer and amine initiator which forms ammonium salt with monomer. Although it has been reported that polar solvents such as DMAc would hinder dethiocarboxylation of NTAs during polymerization^52^, we successfully carry out the homopolymerization of CPG-NTA in DMAc with relatively high yields of 72–91% (Samples 1–4). All polymerizations reach full conversion (>99%) except Sample 4 (97%) at the end of 2-day reaction. Degrees of polymerization (DPs) are predictable depending on the feed ratios of CPG-NTA and initiator. The MWs obtained from NMR (Fig. 1b) and SEC analyses (Fig. 1c) keep consistent and the dispersities (*Đ*_SEC_) are lower than 1.12. Narrow symmetrical monomodal in size exclusion chromatography (SEC) traces of polyCPG products (Sample 1–4 in Fig. 1c) indicate good controllability of polymerization. When the feed ratio rises, slight decline of yield is observed, which is resulted from low concentration of amine, propagation reaction competed by impurities and side reaction in polar solvent. The polymerization is also examined in nonpolar solvent chloroform (Sample 5). The product, precipitated oligomer, is insoluble in chloroform due to pendant carboxyl groups and polymerization terminates at low conversion.

**Table 1:** Polymerization of CPG-NTA and CBG-NTA initiated by neopentylamine^a^.

Sample	Monomer	[M]_0_/[I]_0_	Conv. %	Yield %	*M*_n theo_^b^ (kg mol^−1^)	DP_NMR_^c^	*M*_n NMR_^c^ (kg mol^−1^)	*M*_n SEC_^d^ (kg mol^−1^)	*Đ*_SEC_^d^
1	CPG-NTA	8	>99	91	1.3	8	1.5	2.8	1.12
2	CPG-NTA	20	>99	86	3.0	18	3.2	5.2	1.08
3	CPG-NTA	50	>99	84	7.3	45	7.8	7.8	1.09
4	CPG-NTA	70	97	73	8.9	58	10.0	9.3	1.09
5^e^	CPG-NTA	20	39	30	1.1	7	1.3	2.6	1.09
6	CBG-NTA	20	>99	80	2.6	11	1.6	1.2	1.48

^a^ Polymerization conditions: [M]_0_ = 0.5 mol L^−1^, 48 h at 60 °C in DMAc except Sample 5.

^b^ Number-average molecular weight (*M*_n_) is calculated by *M*_n theo_ = [M]_0_/[I]_0_ × yield × MW of repeat units + MW of initiator.

^c^ Determined by ^1^H NMR.

^d^ Determined by SEC.

^e^ Polymerization conditions: [M]_0_ = 0.5 mol L^−1^, 24 h at 58 °C in chloroform.

The products have been characterized by NMR and all signals in ^1^H NMR and ^13^C NMR spectra (Fig. 1b and Supplementary Fig. 4) are fully assigned to the corresponding atoms of polyCPG. In ^13^C NMR spectrum (Supplementary Fig. 4), the signal of carboxylic acid is found at 174.60 ppm while those of amide backbone are observed at 169.24 ppm. The matrix-assisted laser desorption ionization-time of flight (MALDI-ToF) MS of polyCPG (Fig. 1e, f, Sample 3) shows a symmetric monomodal profile, which reveals neopentyl and amino end groups with CPG repeating units. In the zoom-in view (Fig. 1e), because of the substitution of proton of carboxyl group at the side chain by potassium cation, a series of weak peaks follow every major peak by a shift of 38 Da. The existence of massive carboxyl groups in polyCPG is thus confirmed. We successfully realize a direct polymerization of amino acid derivative bearing unprotected acidic pendent group.

CBG-NTA is another NNTA with unprotected carboxyl group (Supplementary Fig. 5, Supplementary Methods). The successful polymerization of CBG-NTA into polyCBG (Sample 6, Supplementary Figs. 6–8) demonstrate that CPG-NTA is not the only NNTA which is capable of polymerizing to acidic polypeptoids. Scientists can translate this system to other NNTAs with unprotected carboxyl groups.

**Fig. 2:**
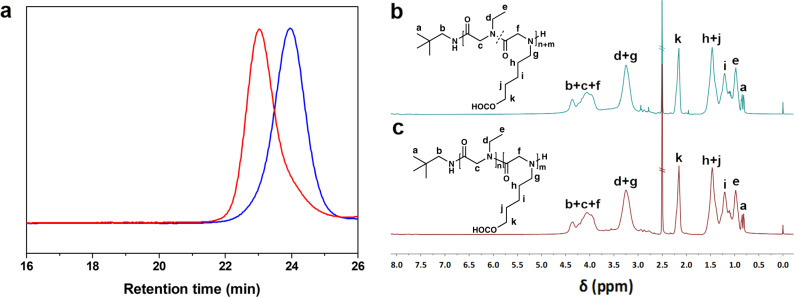
Characterization of products obtained from block-copolymerization of CPG-NTA with NEG-NTA. SEC traces **a** of first block polymer polyNEG (blue) and block copolymer poly(NEG-*b*-CPG) (Sample 8, red), and ^1^H-NMR spectra of poly(CPG-*r*-NEG) (Sample 7) (**b**) and poly(NEG-*b*-CPG) (Sample 8) (**c**).

To further investigate the polymerization behaviors of CPG-NTA and incorporate it into polymerizations of other amino acids, we carry out both random and block copolymerizations of CPG-NTA with NEG-NTA (Table 2). The random one produces poly(CPG-*r*-NEG) with composition close to the feed ratio and symmetrically monomodal SEC trace (Supplementary Fig. 9). By sequential feed of NEG-NTA and CPG-NTA monomers, poly(NEG-*b*-CPG) block copolymer is successfully obtained. The block polymerization reaches decent yield (>82%). The obvious peak shifts observed in SEC (Fig. 2a) from first block polymer indicate that most polyNEG chains are capable of further initiating the polymerization of CPG-NTA monomer in DMAc. Diffusion ordered spectroscopy spectrum (DOSY, Supplementary Fig. 10) of block copolymer (Sample 8) also excludes the existence of neither polyNEG nor polyCPG homopolymer. The structures of both copolymers are confirmed by ^1^H NMR spectra (Fig. 2b, c). The success in preparation of copolymer of CPG-NTA reveals its ability to build complicated polymers for certain applications.

**Table 2:** Copolymerization of CPG-NTA with NEG-NTA initiated by neopentylamine.

Sample	Feed molar ratio	Yield %	Polymer composition^c^	*M*_n SEC_^d^ (kg mol^−1^)	*Đ*_SEC_^d^
7^a^	[CPG]_0_/[NEG]_0_/[I]_0_ = 18:20:1	83	poly(CPG_19_-*r*-NEG_20_)	6.5	1.15
8^b^	[NEG^1st^]_0_/[CPG^2nd^]_0_/[I]_0_ = 24:21:1	82	poly(NEG_18_-*b*-CPG_18_)	4.9	1.15

^a^ Polymerization conditions: [M]_0_ = 0.5 mol L^−1^ in total, 48 h at 60 °C in DMAc.

^b^ Polymerization conditions: [M]_0_ = 0.5 mol L^−1^ for NEG-NTA and CPG-NTA, respectively at 60 °C in DMAc, 24 h for each block. Monomer molar equivalent amount of acetic acid is added to promote the polymerization.

^c^ Determined by ^1^H NMR.

^d^ Determined by SEC.

### Preparation and polymerization of IDA-NTA

**Fig. 3:**
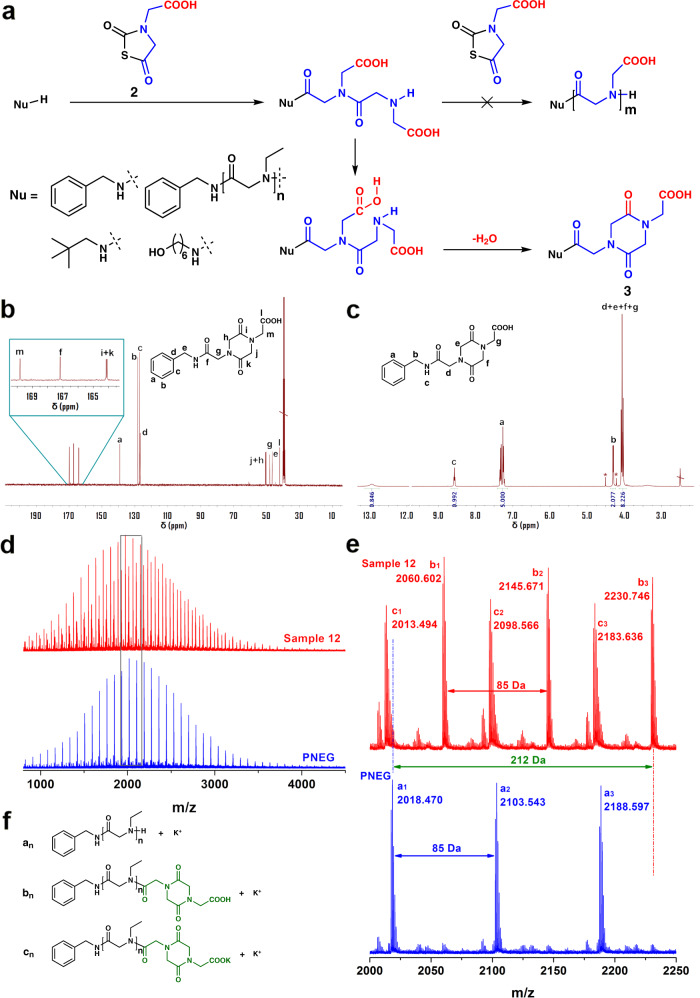
Capping reaction of IDA-NTA and characterization of products. Scheme of reaction between IDA-NTA and benzylamine or polyNEG (**a**). ^13^C NMR spectrum of product obtained from reaction between IDA-NTA and benzylamine with feed-ratio of 4 (**b**). ^1^H NMR spectrum of product described above (\ DMSO, * IDA-NTA) (**c**). MALDI-ToF MS of macroinitiator polyNEG (blue) and product of reaction (red) in Sample 12, Supplementary Table 1 (**d**) with zoom-in views (**e**) and the corresponding chemical structures (**f**).

IDA is an easily accessible *N*-substituted glycine bearing two symmetrical carboxyl groups. Ester protected IDA-NTA has been synthesized to be bioactive gas donor^54^. Taking advantage of its symmetric carboxyl groups, we successfully synthesize IDA-NTA (**2** in Fig. 3a, Supplementary Fig. 11, Supplementary Methods) with high yields. It is characterized by ^1^H NMR (Supplementary Fig. 12a), ^13^C NMR (Supplementary Fig. 12b) and ESI-MS (Supplementary Fig. 13) spectra. Its ^13^C NMR and ^1^H NMR spectra present three carbonyl signals and a signal of carboxyl proton at downfield, respectively, indicating the presence of unprotected carboxyl group similar to that in CPG-NTA.

Ring-opening reaction of IDA-NTA is investigated in various solvents (Fig. 3a, Samples 9–11 in Supplementary Table 1). Unlike CPG-NTA, the polymerization of IDA-NTA results in extremely low conversion of 4–10% in both nonpolar (Tetrahydrofuran (THF) and chloroform, Samples 9 and 10) and polar solvents (DMAc, sample 11). The ^1^H NMR spectra and SEC traces (Supplementary Figs. 14 and 15) of the products (Sample 9 and 10) exclude the existence of polymer. Although the signals of IDA repeating units are found in the ^1^H NMR spectrum of product precipitated from DMAc (sample 11, Supplementary Fig. 14c), the absence of polymer signal peak in its SEC trace (Supplementary Fig. 15) suggests only oligomerization of IDA-NTA. To figure out the reason behind the termination of IDA-NTA polymerization, we treat IDA-NTA with benzylamine at a feed ratio of 4 to analyze the chain end of oligoIDA obtained as a white powder. Four signals at 169.82, 167.18, 164.15 and 164.09 ppm in ^13^C NMR spectrum (Fig. 3b) are ascribed to four carbonyl groups. Moreover, two of them (C^i^ and C^k^) have very similar chemical environment indicating the existence of a six-membered ring composed of two identical *N*-substituted amide groups (**3** in Fig. 3a). DP of IDA, 2.06, slightly higher than 2, is calculated by the integral ratio between IDA repeating units (H^d+e+f+g^) and phenyl initiator residue (H^a^) in ^1^H NMR spectrum (Fig. 3c). The ESI-MS of product (Supplementary Fig. 16) confirms it as a cyclized IDA dimer capped by benzylamine. A small amount of species with a DP of 3 is also observed in ESI-MS indicating overwhelming cyclization but propagation. The amidation reaction between amine and the carboxyl side group terminates propagation and transforms amine end groups into carboxyl end group with decent yield (89%) and quantitative conversion (99%) of benzylamine calculated from signal of amide proton (H^c^) in Fig. 3c. The absence of signal ascribed to amine-ended species in ESI-MS (Supplementary Fig. 16) also confirms the full conversion of amines. The reaction between IDA-NTA and neopentylamine also results in the corresponding end-capped product with a precise DP of 2 characterized by NMR spectra (Supplementary Fig. 17).

PolyNEG_20_ (Supplementary Table 1, sample 12) is applied to validate the effectiveness of IDA-NTA as a capping reagent to transform amines into carboxylic acids. Both the polyNEG macroinitiator and the isolated product are analyzed by MALDI-ToF MS in Fig. 3d–f. No obvious shift from polyNEG_20_ to high MW is detected (Fig. 3d). In the zoom-in view (Fig. 3e), the disappearance of polyNEG (**a**_**n**_) populations indicates the consumption of polyNEG by initiating ring-opening reaction of IDA-NTA. At the meantime, the populations with an m/z increase of 212 Da appear as **b**_**n**_ in the product spectrum. They are ascribed to polyNEG capped by 1,4-dicarboxymethylpiperazine-2,5-dione as a cyclic IDA dimer. The signals **c**_**n**_ with a shift of 38 Da from **b**_**n**_ provide evidence of potassium carboxylate end group converted from carboxylic acid.

Our previous work proves the tolerance of hydroxyl groups in NTA polymerizations. IDA-NTA termination is a regio-selective way to realize capping reaction of polymers or molecules to transform amine groups into carboxyl groups. The same capping reaction described is applied to 6-amino-1-hexanol resulting in high yield (99%). In ^1^H and ^13^C NMR spectra (Supplementary Fig. 18), the proton and carbon signals of the methylene group near hydroxyl group keep unchanged indicating the absence of capping reaction at the hydroxyl group. No ester group is detected supporting quantitative regio-selectivity of the capping reaction distinguishing amino group from hydroxyl one. The structure is also confirmed by ESI-MS (Supplementary Fig. 19) and no signal of amine-ended species is found. Instead of a monomer, IDA-NTA is demonstrated as an efficient regio-selective reagent to modify primary and secondary amino groups into carboxylic acids in one step with quantitative conversion. Though hydroxyl groups are common competent nucleophiles when using cyclic acid anhydride as capping reagent of amines, IDA-NTA approach has an advantage to tolerate them in the synthesis of carboxyl-ended functional polymers.

### DFT calculation and NNTA with various linkers

DFT calculation is applied to figure out the difference between CPG-NTA and IDA-NTA polymerizations. All possible condensation routes of IDA (routes **A** and A*) and CPG ends can be processed via two dehydration pathways, i.e., carbonyl addition-dehydration (routes **A**, **B** and **C**) and directly dehydration (routes A*, B* and C*) as shown in Supplementary Fig. 20 (Supplementary Data 1). The condensation of CPG end unit can occur with the last unit (route **B**) or the nearby one (**C**). Direct dehydration step, i.e., A* and C* are observed with lower *ΔG* in route **A** and **C** rather than carbonyl addition-dehydration steps since the formation of six- or ten-membered ring is enthalpy preferred as shown in Supplementary Table 2. Route A* for IDA end is confirmed with the lowest Δ*G* (37.6 kcal mol^−1^) among the three, which indicates that IDA end group is proved with stronger tendency to dehydration than CPG with Δ*G* of 42.4 and 51.1 kcal mol^−1^ (routes **B** and C*).

NNTAs with intermediate-length linkers between CPG-NTA and IDA-NTA are checked. The successful polymerization of CBG-NTA (Sample 6) shows that NNTAs with unprotected carboxyl group can polymerize into the corresponding acidic polypeptoids. IDA-NTA is a special monomer in which the formation of six-member ring dimer prevents the polymerization according to experiments and calculation results. Unfortunately, we failed to synthesize *N*-carboxypropyl glycine NTA and *N*-carboxyethyl glycine NTA due to the competitive cyclization to seven- and six-member ring NNTAs, respectively. CPG-NTA is the best candidate to synthesize polypeptoids with pendant carboxyl groups among all checked NNTAs due to its good controllability and cheap origin.

### Physical property and application of polyCPG

**Fig. 4:**
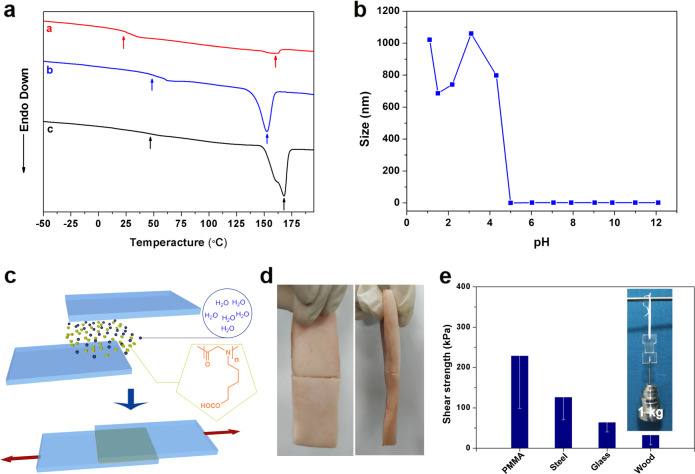
Physical property test and adhesion experiment of polyCPGs. DSC profiles of Samples 2 (a, red) 3 (b, blue) and 4 (c, black) (**a**). pH-responsive solubility of polyCPG (Sample 2) in water (**b**). Illustration of adhesion procedure (**c**). Adhesion experiment of polyCPG on swine skin tissue (**d**). Shear strength of polyCPGs on different materials (**e**). The inset image illustrates the adhesive behavior of polyCPGs on PMMA.

Thermogravimetric analysis (TGA) and differential scanning calorimetry (DSC) analyses have been applied to investigate the thermal property of polyCPG. The TGA trace (Supplementary Fig. 21) of polyCPG (Sample 3) shows that the decomposition temperature of 245 °C is close to that of other polypeptoids including polysarcosine and poly(*N*-butyl glycine)^45^ because of the same amide backbones. In the DSC profiles (Fig. 4a) of polyCPG (Samples 2–4) with various DPs, three samples show glass transition temperatures (*T*_g_s) of 19 °C, 54 °C and 43 °C, respectively. The melting temperatures (*T*_m_s) among all profiles indicate crystal phase in polyCPG samples. *T*_m_s of three samples are 160 °C, 152 °C and 168 °C with the corresponding melting enthalpies (Δ*H*_m_s) of 3.21 J g^−1^, 20.92 J g^−1^ and 47.36 J g^−1^, respectively. As DPs of polyCPGs increase, Δ*H*_m_s rise obviously due to the growth of crystal phase in the polymer samples. A series of peak signals observed in XRD pattern of Sample 3 (Supplementary Fig. 22) further demonstrate that polyCPG crystallizes and forms ordered structures as other polypeptoids with long side chains^38^.

The solubility of polyCPG is investigated by dissolving polyCPG (Sample 2) in various solvents (Supplementary Table 3, Supplementary Methods). Due to the high polarity of carboxyl groups at the side chain, polyCPG is only able to dissolve in polar solvents including *N,N*-dimethylformamide (DMF), DMSO and alcohols, except acetonitrile and benzonitrile which are common solvents for many polypeptoids. Because of carboxyl groups, solubility of polyCPG in water is pH-responsive. A scan of solubility of polyCPG in water with different pHs by dynamic light scattering (DLS, Fig. 4b) shows that polyCPG dissolves well in alkali water giving very weak DLS signals. When pH decreases from 5 to 4.3, the solution turns turbid as polyCPG precipitates with irregular particle diameters. pH-responsiveness of polyCPG is a useful property for its further application in aqueous systems.

Due to amide backbone and carboxyl side groups, polyCPG samples are able to form hydrogen bonds between them or with other substances. While alkyl polypeptoids including polyNEG and polysarcosine show no adhesivity, the abundant hydrogen bonds enable polyCPG to serve as a surface adhesive material similar to other carboxyl group-containing polymers^56^. PolyCPG powders and drops of water are deposited together on material surfaces and followed by rubbing or heating to activate the chain movement. It becomes pretty sticky to adhere two slices of substances together. Subsequent loss of solvent allows polyCPG to form a strong film between the two slices adhered (Fig. 4c). A weight of 1 kg can be hung under two slices of adhered poly(methyl methacrylate) (PMMA) after being dried overnight (Fig. 4e, inset image). We investigate the adhesion property of polyCPG by measuring the shear strength of the slices pulled slowly apart in the direction parallel to the adhered surfaces (Fig. 4c). The polypeptoid exhibits decent adhesive capability (33 to 229 kPa) to various materials including PMMA, stainless steel, wood and glass without chemical cross-linking (Fig. 4e). Figure 4d shows that pig skin tissue can be adhered immediately by polyCPG without being dried. The separated slices can be adhered again followed the methods described above and polyCPG is able to be recycled by washing it from slice surfaces with ethanol. PolyCPG is a promising candidate as a biocompatible adhesive material.

## Methods

### Synthesis of amino acids and NTAs

CPG hydrochloride were prepared according to the reported protocol^57^. The prepared CPG hydrochloride (28.8 g, 0.1276 mmol) and *S*-ethoxythiocarbonyl mercaptoacetic acid (23.0 g, 0.1276 mol) were dissolved in 300 mL aqueous solution of NaOH (20.5 g, 0.5104 mol). The mixture was stirred for 3 days at room temperature. Then it was acidified by concentrated hydrochloric acid and extracted with ethyl acetate. The organic phase was washed with aqueous critic acid (5 wt%) and brine. The solvent was evaporated under reduced pressure after dried over Na_2_SO_4_. The concentrated liquid was purified by column (ethyl acetate: petroleum ether = 1:2) and a white powder was obtained by recrystallization in ethanol and water (7.2 g, 2.596 mmol). Then the powder was dissolved in 300 mL ethyl acetate. 4.5 mL PBr_3_ (about 12.7 g, 0.0469 mmol) was added dropwise to the solution in 0 °C ice bath in 15 min. After stirred for 1 h at room temperature, the mixture was washed by water and brine for 3 times, respectively, and then dried over MgSO_4_. A white powder was obtained by recrystallization in ethyl acetate and petroleum ether (4.7 g, yield 16%) and to be stored under an argon atmosphere.

The synthesis of CBG-NTA and IDA-NTA followed similar procedure described above. The experimental details and monomers characterization are included in Supplementary Methods.

### Polymerization of NTAs

All polymerizations and end-capping reactions were carried out using Schlenk technique, and all reaction vials were flame dried and purged with argon.

As a typical polymerization, CPG-NTA (170.2 mg, 0.7359 mmol) was dissolved in 1.2 mL DMAc followed by 0.28 mL neopentylamine solution in DMAc (0.05208 mmol mL^−1^). The reaction was carried out at 60 °C for 48 h. After precipitation from diethyl ether and centrifugation, the product was collected and dried under vacuum to a constant weight (107 mg, 84%).

As a typical polymerization or end-capping reaction of IDA-NTA, IDA-NTA (0.3098 g, 1.815 mmol) was dissolved in 2.8 mL THF followed by 0.50 mL benzylamine solution in THF (0.1812 mmol mL^−1^). It was incubated in 60 °C for 24 h. After precipitation from diethyl ether and centrifugation, the product was dried under vacuum to constant weight (9 mg, 4%).

### Adhesive experiment

A polyCPG sample (25 mg) was deposited on a slice with a 20 mm × 20 mm area followed by 20 μL deionized water. By rubbing the polymer swelled with another slice, they were adhered. The samples were let stand still overnight at room temperature to dry before tensile measurement except adhered swine skin tissue. The slices were drawn by tensile tester in the direction parallel to the adhered surface at a rate of 5 mm min^−1^. The shear strength was calculated from the maximum force divided by the area of adhered surface. The experiments for every material are repeated for three times and standard errors are calculated.

### Calculation details

All geometries of transition states (TSs) and intermediates were optimized under tight criteria using M06-2X^58^/6-31G(d,p) with DFT-D3 correction^59^. Frequency calculations were employed to confirm that the intermediates and TSs had zero and one imaginary frequency, respectively. The reaction pathway of all TS was checked by intrinsic reaction coordinate (IRC)^60^. Thermal correction to Gibbs free energies was obtained at 298.2 K and 1.013 × 10^5^ Pa. All calculations were performed using Gaussian 16 program as we reported before^61–63^.

## Supplementary information

Supplementary Information

Description of Additional Supplementary Files

Supplementary Data 1

## Data Availability

The data used in this study are available from the corresponding authors upon reasonable request.
