# Supplementary material for: Direct N-substituted N-thiocarboxyanhydride polymerization towards polypeptoids bearing unprotected carboxyl groups
Source: Commun Chem. 2020 Oct 28;3:144. doi: 10.1038/s42004-020-00393-y (PMC9814353; doi:10.1038/s42004-020-00393-y)
Supplement: Supplementary file 1 — Supplementary Information [file 42004_2020_393_MOESM1_ESM.docx]

**Supplementary Information**

**Direct *N*-substituted *N*-thiocarboxyanhydride polymerization towards polypeptoids bearing unprotected carboxyl groups**

Botuo Zheng,^1,2^ Tianwen Bai,^2^ Jun Ling,^2,^* Jihong Sun ^1,3,^*

^1^ Department of Radiology, Sir Run Run Shaw Hospital, School of Medicine, Zhejiang University, Hangzhou 310016, China.

^2^ MOE Key Laboratory of Macromolecular Synthesis and Functionalization, Department of Polymer Science and Engineering, Zhejiang University, Hangzhou 310027, China.

^3^ Innovation Center for Minimally Invasive Techniques and Devices, Zhejiang University, Hangzhou 310016, China.

CONTENTS

1. Supplementary Methods **S4**

1.1 Characterization **S4**

1.2 Materials **S4**

1.3 Synthesis and Characterization **S5**

1.3.1 Synthesis of CPG and CPG-NTA **S5**

1.3.2 Synthesis and polymerization of CBG-NTA **S5**

1.3.3 Synthesis of IDA-NTA **S6**

1.3.4 Solubility Test **S6**

2. Supplementary Figures and Tables **S7**

**Supplementary Figure 1.** Synthesis of CPG-NTA. **S7**

**Supplementary Figure 2.** ^1^H and ^13^C NMR spectra of CPG-NTA. **S7**

**Supplementary Figure 3.** Mass spectrum of CPG-NTA. **S8**

**Supplementary Figure 4.** ^13^C NMR spectrum of polyCPG. **S8**

**Supplementary Figure 5.** Synthesis of CBG-NTA. **S9**

**Supplementary Figure 6.** ^1^H and ^13^C NMR spectra of CBG-NTA. **S9**

**Supplementary Figure 7.** ^1^H NMR spectrum of polyCBG (Sample 6). **S9**

**Supplementary Figure 8.** SEC trace of polyCBG (Sample 6). **S10**

**Supplementary Figure 9.** SEC trace of poly(CPG-*r*-NEG) (Sample 7). **S10**

**Supplementary Figure 10.** DOSY spectrum of poly(NEG-*b*-CBG) (Sample 8). **S11**

**Supplementary Figure 11.** Synthesis of IDA-NTA. **S11**

**Supplementary Figure 12.** ^1^H and ^13^C NMR spectra of IDA-NTA. **S12**

**Supplementary Figure 13.** Mass spectrum of IDA-NTA. **S12**

**Supplementary Table 1.** Polymerization of IDANTA. **S13**

**Supplementary Figure 14.** ^1^H NMR spectra of the products obtained from IDA-NTA polymerization. **S13**

**Supplementary Figure 15.** SEC traces of the products obtained from IDA-NTA polymerization: Samples 9-11. **S14**

**Supplementary Figure 16.** Mass spectrum of cyclized IDA dimer initiated by benzylamine. **S14**

**Supplementary Figure 17** ^1^H and ^13^C NMR spectra of cyclic IDA dimer initiated by neopentylamine. **S15**

**Supplementary Figure 18.** ^1^H and ^13^C NMR spectra of cyclic IDA dimer initiated by 6-amino-1-hexanol. **S15**

**Supplementary Figure 19.** Mass spectrum of cyclic IDA dimer initiated by 6-amino-1-hexanol. **S16**

**Supplementary Figure 20.** Proposed mechanism based on DFT calculations with ΔG of each step. **S17**

**Supplementary Table 2.** Gibbs free energy barrier value of all three possible routes. **S18**

**Supplementary Figure 21.** The TGA profile of Sample 4. **S18**

**Supplementary Figure 22.** XRD trace of polyCPG (Sample 3). **S19**

**Supplementary Table 3.** Solubilities of polyCPG in common organic solvents. **S19**

3. Supplementary References **S20**

- **Supplementary Methods**

**1.1 Characterization.**

Nuclear magnetic resonance (NMR) spectra were recorded on a Bruker Avance DMX 400 spectrometer (^1^H: 400 MHz; ^13^C: 100 MHz). DMSO-*d*_6_ and CDCl_3_ were used as solvent with tetramethylsilane (TMS) as internal reference. Size exclusion chromatography (SEC) instrument consisted of a Waters 1515 isocratic HPLC pump, a Waters 2414 interferometric refractometer (RI) and two Shodex KF series columns. Hexafluoroisopropanol (HFIP) containing 3 mg mL^-1^ potassium trifluoroacetate was used as the eluent with a flow rate of 0.8 mL min^-1^ at 40 °C. Poly(methyl methacrylate) standards with narrow polydispersities were used for MW calibration. Matrix-assisted laser desorption ionization-time of flight (MALDI-ToF) mass spectra were collected on a Bruker UltraFLEX MALDI-ToF in reflector mode with potassium trifluoroacetate as the cationic agent and 2, 5-dihydroxybenzoic acid (DHB) as the matrix. Mechanical properties of the material slices adhered were measured using a commercial tensile tester (Instron 3343, United States) with a crosshead speed of 5 mm min^-1^. Thermogravimetric analysis (TGA) was performed on a TA Q50 instrument. Samples were heated to 600 °C at a rate of 10 °C min^-1^. Differential scanning calorimetry (DSC) analysis were tested on a TA Q20 instrument. Samples were heated to 210 °C at a rate of 10 °C min^-1^ under nitrogen purge, cooled to -80 °C min^-1^ at a rate of -10 °C min^-1^ and subjected to a second scan. Powder X-Ray diffraction (XRD) data were collected on a Rigaku Ultimate-IV X-ray diffractometer operating at 40 kV/30 mA using the Cu Kα line (λ= 1.5418 Å). Powder was measured over the range of 5-90° in 5 ° min^-1^ steps. pH values were measured by an INESA PHSJ-3F pH meter in continuous mode. Dynamic light scattering (DLS) measurements were carried out on a particle size analyzer (Zetasizer Nano Series, Malvern Instruments) at 25 °C. Electrospray ionization mass spectra (ESI-MS) were recorded on an LCD Deca XP Max ion trap mass spectrometer with APCI ion source.

**1.2 Materials.**

Glyoxylic acid monohydrate (98%, Energy Chemical, China), glyoxylic acid (50 wt% aqueous solution, Energy Chemical, China), phosphorus tribromide (99%, Energy Chemical, China), neopentylamine (98%, Energy Chemical, China), ethylamine (68.0-72.0 wt%, Energy Chemical, China), 6-aminocaproic acid (98%, Energy Chemical, China), 5-aminovaleric acid (98%, Energy Chemical, China), 6-amino-1-hexanol (97%, J&K, China), IDA (97%, Energy Chemical, China), dimethylacetamide (DMAc, 99.8%, super dry, J&K, China) and tetrahydrofuran (THF, 99.9%, super dry, J&K, China) were used as received. Chloroform (AR, Sinopharm Chemical Reagent, China) was distilled before use. Benzylamine was stirred over CaH_2_ and followed by distillation under reduced pressure. NEG-NTA was synthesized according to procedures reported in our previous work.^1^

- **Synthesis and characterization**

**1.3.1 Synthesis of CPG and CPG-NTA**

CPG hydrochloride was prepared according to the reported protocol.^2^ 6-Aminocaproic acid (15.6 g, 0.119 mol) was dissolved in 300 mL water followed by glyoxylic acid monohydrate (55.2 g, 0.321 mol). The solution was stirred overnight. After acidified by excess concentrated hydrochloric acid (50 mL), the solution was refluxed for 12 h. CPG was precipitated from a mixture of methanol and diethyl ether after concentrated and a sticky yellow solid was obtained (16.2 g, yield 60%). ^1^H NMR (DMSO-*d*_6_) δ: 1.28 ppm (m, 2H), 1.52 ppm (m, 2H), 1.63 ppm (m, 2H), 2.30 ppm (t, 2H), 2.87 ppm (s, 2H), 3.82 ppm (s, 2H), 9.25 ppm (s, 2H).

The synthesis of CPG-NTA are described in text and the monomer is characterized by NMR and mass spectrum. ^1^H NMR (CDCl_3_) δ: 1.41 ppm (m, 2H), 1.68 ppm (m, 4H), 2.39 ppm (t, 2H), 3.54 ppm (t, 2H), 4.19 ppm (s, 2H). ^13^C-NMR (CDCl_3_) δ:194.07 ppm (-SCOCH_2_-), 179.40 ppm (-CH_2_COOH), 164.93 ppm (-SCON-), 59.82 ppm (-COCH_2_N-), 43.95 ppm (-NCH_2_CH_2_-), 33.66 ppm (-CH_2_CH_2_COOH), 27.10 ppm (-NCH_2_CH_2_CH_2_-), 25.94 ppm (-CH_2_CH_2_CH_2_COOH), 24.05 ppm (-CH_2_CH_2_CH_2_CH_2_CH_2_-). ESI-MS of CPG-NTA: m/z calculated for C_9_H_12_NO_4_S^-^: 230.05 [M-H]^-^, found: 230.09.

**1.3.2 Synthesis and polymerization of CBG-NTA**

CBG hydrochloride was prepared according to the reported protocol.^2^ 5-Aminovaleric acid (10.3 g, 0.0999 mol) was dissolved in 200 mL water followed by 50 wt % glyoxylic acid aqueous solution(35.6 g, 0.2404 mmol). The solution was stirred overnight. After acidified by excess concentrated hydrochloric acid (38 mL), the solution was refluxed for 12 h. CPG was precipitated from a mixture of methanol and diethyl ether after concentrated and a sticky yellow solid was obtained (11.0 g, yield 52%). ^1^H NMR (DMSO-*d*_6_) δ: 1.53 ppm (m, 2H), 1.64 ppm (m, 2H), 2.24 ppm (t, 2H), 2.90 ppm (t, 2H), 3.83 ppm (s, 2H), 9.17 ppm (s, 2H).

The prepared CBG hydrochloride (5.3 g, 0.025 mol) and *S*-ethoxythiocarbonyl mercaptoacetic acid (6.3 g, 0.035 mol) were dissolved in 150 mL aqueous solution of NaOH (4.4 g, 0.11 mol). The mixture was stirred for 3 days at room temperature. Then it was acidified by concentrated hydrochloric acid and extracted with chloroform. The organic phase was washed with brine. The solvent was evaporated under reduced pressure after dried over Na_2_SO_4_. The concentrated liquid was purified by column (ethyl acetate: petroleum ether = 1:1) and a colorless liquid was obtained. Then the liquid was diluted in 25 mL chloroform. 0.4 mL PBr_3_ (about 1.1 g, 0.0469 mmol) was added dropwise to the solution in 0 ºC ice bath in 15 min. After stirred for 1 h at room temperature, the mixture was washed by aqueous critic acid (5 wt%) and brine for 3 times, respectively, and then dried over MgSO_4_. A white powder was obtained by recrystallization in ethyl acetate and petroleum ether (0.3 g, yield 5%) and to be stored under an argon atmosphere. ^1^H NMR (CDCl_3_) δ: 1.69 ppm (m, 4H), 2.44 ppm (t, 2H), 3.55 ppm (t, 2H), 4.20 ppm (t, 2H). ^13^C NMR (CDCl_3_) δ:194.07 ppm (-SCOCH_2_-), 178.73 ppm (-CH_2_COOH), 165.23 ppm (-SCON-), 59.84 ppm (-COCH_2_N-), 43.81 ppm (-NCH_2_CH_2_-), 33.19 ppm (-CH_2_CH_2_COOH), 26.80 ppm (-NCH_2_CH_2_CH_2_-), 21.59 ppm (-CH_2_CH_2_CH_2_COOH).

**1.3.3 Synthesis of IDA-NTA**

The synthesis of IDA-NTA is the same as CPG-NTA by using IDA as the starting reagent. White crystals were obtained as the product (yield 20.3 %). ^1^H-NMR (DMSO-*d*_6_) δ: 4.21 ppm (s, 2H), 4.50 ppm (s, 2H). ^13^C-NMR (DMSO-*d*_6_) δ: 194.32 ppm (-SCOCH_2_-), 169.06 ppm (-CH_2_COOH), 164.92 ppm (-SCON-), 60.74 ppm (-COCH_2_N-), 44.57 ppm (-NCH_2_COOH). ESI-MS of IDA-NTA: m/z calculated for C_5_H_4_NO_4_S^-^: 173.99 [M-H]^-^, found: 173.98.

**1.3.4 Solubility test**

For pH-responsive aggregation test, 138 mg polyCPG powder was suspended in 65 mL deionized water followed by 3 mL NaOH (1 M) solution to prepare a yellow 0.2% polyCPG solution. After the full dissolution of polyCPG, the solution was filtered by a 0.45 μm aqueous filter to remove dusts and insoluble impurities. By adding HCl (1 M) dropwise, the solution was acidified. Approximately 5 mL solution was isolated to a test tube every time the pH decreased about 1. Then the polyCPG solutions with gradient pHs were subjected to DLS test to determine the particle sizes in the solution.

- **Supplementary Figures and Tables**

**Supplementary Figure 1.** Synthesis of CPG-NTA.

**Supplementary Figure 2.** ^1^H (a) and ^13^C (b) NMR spectra of CPG-NTA. (* CHCl_3_, ** TMS).

**Supplementary Figure 3.** Mass spectrum of CPG-NTA, m/z calculated for C_9_H_12_NO_4_S^-^: 230.05 [M-H]^-^, found: 230.09.

**Supplementary Figure 4.** ^13^C NMR spectrum of polyCPG (Sample 2, \\ diethyl ether).

**Supplementary Figure 5.** Synthesis of CBG-NTA.

**Supplementary Figure 6.** ^1^H (a) and ^13^C (b) NMR spectra of CBG-NTA.

**Supplementary Figure 7.** ^1^H NMR spectrum of polyCBG (Sample 6) (\\ DMSO).

**Supplementary Figure 8.** SEC trace of polyCBG (Sample 6).

**Supplementary Figure 9.** SEC trace of poly(CPG-*r*-NEG) (Sample 7).

**Supplementary Figure 10.** DOSY spectrum of poly(NEG-*b*-CBG) (Sample 8). log***D*** = -10.41 m^2^ s^-1^.

**Supplementary Figure 11.** Synthesis of IDA-NTA

**Supplementary Figure 12.** ^1^H (a) and ^13^C (b) NMR spectra of IDA-NTA (^*^ H_2_O, ** DMSO). Three carbonyl signals and a signal of carboxyl proton at downfield indicate the presence of unprotected carboxyl group similar to that in CPG-NTA.

**Supplementary Figure 13.** Mass spectrum of IDA-NTA, m/z calculated for C_5_H_4_NO_4_S^-^: 173.99 [M-H]^-^, found: 173.98.

**Supplementary Table 1.** Polymerization of IDA-NTA. *^a^*

| Sample | Initiator | [M]_0_/[I]_0_ | Solvent | Conversion% | DP _NMR_ *^b^* |
| --- | --- | --- | --- | --- | --- |
| 9 | benzylamine | 20/1 | THF | 4 | 2 |
| 10 |  |  | Chloroform | 10 | 4 |
| 11 |  |  | DMAc | 8 | - |
| 12 | polyNEG_20_ | 15/1 | THF | 6 | 2 |

*^a^* Polymerization conditions: [M]_0_ = 0.5 mol L^-1^, 24 h at 60 °C. *^b^* Determined by ^1^H NMR.

**Supplementary Figure 14.** ^1^H NMR spectra of the products obtained from IDA-NTA polymerization: Samples 9 (a), 10 (b) and 11(c) (^*^ DMSO, ** H_2_O).

**Supplementary Figure 15.** SEC traces of the products obtained from IDA-NTA polymerization: Samples 9 (a), 10 (b) and 11(c).

**Supplementary Figure 16.** Mass spectrum of cyclized IDA dimer initiated by benzylamine.

**Supplementary Figure 17** ^1^H (a) and ^13^C (b) NMR spectra of cyclic IDA dimer initiated by neopentylamine (\ DMSO, \\ water).

**Supplementary Figure 18.** ^1^H (a) and ^13^C (b) NMR spectra of cyclic IDA dimer initiated by 6-amino-1-hexanol (\ DMSO)

**Supplementary Figure 19.** Mass spectrum of cyclic IDA dimer initiated by 6-amino-1-hexanol.

**Supplementary Figure 20.** Proposed mechanism based on DFT calculations with ΔG (kcal mol^-1^) of each step.

**Supplementary Table 2.** Gibbs free energy barrier value (kcal mol^-1^) of all three possible routes.

| Route | Ring size | The higher ΔG of TS1 and TS2 | ΔG of TS1_H_2_O | Favored |
| --- | --- | --- | --- | --- |
| A | 6 | 45.0 (**TS2**) | 37.6 | Directly dehydration (route A*) |
| B | 7 | 42.4 (**TS1**) | 45.8 | Carbonyl addition-dehydration  (route B) |
| C | 10 | 58.0 (**TS1**) | 51.1 | Directly dehydration (route C*) |

**Supplementary Figure 21.** The TGA profile of Sample 4.

**Supplementary Figure 22.** XRD pattern of polyCPG (Sample 3). The degree of crystallinity calculated is 22.9 %.

**Supplementary Table 3.** Solubilities of polyCPG in common organic solvents. *^a^*

| Solvent | Solubility | Solvent | Solubility |
| --- | --- | --- | --- |
| acetone | - | ethanol | + |
| acetonitrile | - | ethyl acetate | - |
| benzonitrile | - | methanol | + |
| chloroform | - | isopropanol | + |
| dichloromethane | - | *N,N*-dimethyl acetamide | + |
| diethyl ether | - | *N,N*-dimethyl formide | + |
| dimethyl sulfoxide | + | *N*-methyl pyrrolidone | + |
| 1,4-dioxane | - | tetrahydrofuran | - |

*^a^* Test by adding approximately 1 mg polymer to 1 mL of various solvents.

- **Supplementary References**

1. Tao, X., Zheng, B., Kricheldorf, H. R. & Ling, J. Are N-substituted glycine N-thiocarboxyanhydride monomers really hard to polymerize? *J. Polym. Sci., Part A: Polym. Chem.* **55**, 404-410 (2017).

2. Tomkinson, N., Gibbs, T. & Boomhoff, M. A Mild and Efficient Method for the One-Pot Monocarboxymethylation of Primary Amines. *Synlett* 1573-1576 (2007).
