# Supplementary material for: Direct N-substituted N-thiocarboxyanhydride polymerization towards polypeptoids bearing unprotected carboxyl groups
Source: Commun Chem. 2020 Oct 28;3:144. doi: 10.1038/s42004-020-00393-y (PMC9814353; doi:10.1038/s42004-020-00393-y)
Supplement: Supplementary file 2 — Description of Additional Supplementary Files [file 42004_2020_393_MOESM2_ESM.pdf]

**Description of Additional Supplementary Files**

File Name: Supplementary Data 1
Description: The optimized molecular geometries by M06-2X/6-31G(d,p) in A, A*, B, B*, C and C* route
